# Hypoxia Integration in the Serological Proteome Analysis Unmasks Tumor Antigens and Fosters the Identification of Anti-Phospho-eEF2 Antibodies as Potential Cancer Biomarkers

**DOI:** 10.1371/journal.pone.0076508

**Published:** 2013-10-10

**Authors:** Marie Grandjean, Alexandra Sermeus, Samuel Branders, Florence Defresne, Marc Dieu, Pierre Dupont, Martine Raes, Mark De Ridder, Olivier Feron

**Affiliations:** 1 UCLouvain, Institut de Recherche Expérimentale et Clinique (IREC), Pole of Pharmacology and Therapeutics (FATH), Brussels, Belgium; 2 UZ Brussel, Vrije Universiteit Brussel, Brussels, Belgium; 3 UCLouvain, Institute of Information and Communication Technologies, Electronics and Applied Mathematics (ICTEAM), Machine Learning Group, Louvain-la-Neuve, Belgium; 4 UNamur, Namur Research Institute for Life Sciences (NARILIS), Research Unit of Cell Biology (URBC), Namur, Belgium; Okayama University, Japan

## Abstract

The expression by tumor cells of proteins with aberrant structure, expression or distribution accounts for the development of a humoral immune response. Autoantibodies (aAb) directed against tumor-associated antigens (TAA) may thus be particularly relevant for early detection of cancer. Serological proteome analysis (SERPA) aims to identify such circulating aAb through the immunoblotting of 2D-separated tumor cell proteins with cancer patient serum and the consecutive MS identification of proteins in reactive spots. This method has the advantage to use post-translationally modified proteins as a source of potential TAA. Here, we applied this strategy by using colorectal tumor cells pre-exposed to hypoxia in order to promote the expression of a pattern of TAA more likely to represent *in vivo* conditions. We used two human HCT116 and HT29 colorectal cancer cell lines exposed for 48 hours to 1% O_2_. Spots positive after immunoblotting of 2D-separated lysates of hypoxic cells with the sera of tumor-bearing mice, were collected and analysed by MS for protein identification. Among the hypoxia-specific immunogenic proteins, we identified a phosphorylated form of eukaryotic translation elongation factor 2 (phospho-Thr56 eEF2). We confirmed the increased phosphorylation of this protein in hypoxic colorectal tumor cells as well as in mouse tumors. Using a specific immunoassay, we could detect the presence of corresponding anti-phospho-Thr56 eEF2 aAb in the serum of tumor-bearing mice (*vs* healthy mice). We further documented that the detection of these aAb preceded the detection of a palpable tumor mass in mice and validated the presence of anti-phospho-Thr56 eEF2 aAb in the serum of patients with adenomatous polyps and colorectal carcinoma. In conclusion, this study validates a phosphorylated form of eEF2 as a new TAA and more generally, provides evidence that integrating hypoxia upstream of SERPA offers a more relevant repertoire of TAA able to unmask the presence of circulating aAb.

## Introduction

The contribution of the tumor microenvironment to cancer progression is nowadays well recognized [1]. Hypoxia is one of these microenvironmental parameters which account for phenotypic changes in tumors [2]–[4]. Low oxygen concentration in tumors arises from an imbalance between the supply and the consumption of oxygen mainly due to the immaturity of the tumor vasculature and the rapid cancer cell proliferation, respectively [5]. In response to tumor hypoxia, tumor cells will slow down their protein synthesis machinery while at the same time, induction of transcription factors such as HIF (hypoxia-inducible factor) will promote specific gene expression programs [6], [7]. Hypoxic tumor cells will thus present a proteomic profile distinct of normoxic tumor cells, with the preferential expression of proteins required to support adaptive mechanisms including those leading to angiogenesis and glycolytic switch [8]–[10]. Interestingly, hypoxia also plays a role in carcinogenesis as a consequence of early tumor cell proliferation on epithelial surfaces which are separated from the underlying blood supply by an intact basement membrane [11]. Also, the link between inflammation and cancer is proposed to integrate the hypoxic environment due to the increased metabolism and cell turnover while microvascular network is not (yet) adapted [12]. Interestingly, in colorectal carcinogenesis, the adenoma-carcinoma sequence was reported to be associated with induction of HIF-1α in premalignant lesions [13] as well as with dysplasia [14]; HIF-2α was also reported to promote progression from adenoma to carcinoma [15].

Although hypoxia is recognized as a hallmark of tumors accounting for changes in the tumor cell phenotype, it has been so far largely underestimated as a source of modulation of the pattern of antigens prone to give rise to an immunogenic response. Tumor-associated antigens (TAA) are described as proteins released by tumor cells or peptides exposed at the surface of tumor cells or antigen-presenting cells by MHC class I and II molecules, respectively [16]–[19]. Mutation, truncation, misfolding, over-expression and ectopic expression of proteins in tumor cells are proposed to account for the immunogenicity of these TAA [20]–[22]. Interestingly, autoantibodies (aAb) directed against these modified proteins represent potential biomarkers for early detection of cancer or even prognosis [23]–[26]. The specificity and stability of antibodies together with a relative ease of detection represent key advantages in comparison with other circulating blood components [19]. The SERPA (SERological Proteome Analysis) technique exploits the separation of protein lysates derived from tumor cells onto two-dimensional gels and the consecutive immunoblotting using sera collected from cancer patients [25], [27], [28].

Here, for the reasons exposed above, we chose to integrate hypoxia as an environmental parameter in the SERPA workflow by pre-incubating colorectal cancer cells in 1% O_2_, in order to unmask TAA absent or undetectable in lysates of normoxic tumor cells. We identified different tumor- and hypoxia-specific antigens including the phosphorylated Thr56 form of the eukaryotic elongation factor 2 (eEF2). A dedicated immunoassay was developed and enabled us to validate phospho-eEF2 as a *bona fide* hypoxia-induced TAA and corresponding aAb as potential cancer biomarkers in mice and humans.

## Methods

### Ethics Statement

All the experiments involving mice and tumor cells received the approval of the *Comité d’Ethique Facultaire* of the *Université catholique de Louvain* (UCL) (approval ID 2012/UCL/MD005); mouse studies were carried out according to national animal care regulations.

All patients were hospitalized at the Universitair Ziekenhuis Brussel (Belgium) and gave written informed consent agreeing with the policy of the hospital. This includes anonymous use of residual body material for scientific research purpose in strict accordance with the Declaration of Helsinki and the Article 20.2 of the belgian Law (19-12-2008) relating to the Procurement and Use of Human Bodily Materials for Human Medical Applications and for Scientific Research. This article of law states that consent to research use of human biological materials is considered to be given if the donor did not communicate an objection to such use. Practically, all patients undergoing colonoscopy undergo a blood analysis for evaluating their hemogram and coagulation; this procedure is mandatory to allow endoscopic resection in case polyps are found. None of the authors was involved in the collections of samples: residual blood samples were collected by a nurse and de-identified by a data manager before shipment to the lab for the strict purpose of the current study.

### Cells

Human colorectal carcinoma HCT 116 and HT29 cell lines were purchased from the American Type Culture Collection (ATCC), stored according to the supplier’s instructions and used within 6 months after resuscitation of frozen aliquots. Both cell lines were routinely cultured in McCoy 5A medium (Invitrogen, Paisley, UK) supplemented with 10% fetal bovine serum and antibiotics. Cells were maintained at 37°C in normoxic (21% O_2_, 5% CO_2_) conditions exposed to hypoxia (1% O_2_, 5% CO_2_) in a Invivo2 500 hypoxic chamber for 48 h (Ruskinn, Belgium).

### Mice

Male 7-week-old male NMRI mice (nu/nu) (Elevage Janvier, Le Genest Saint-Isle, France) were subcutaneously injected with 2.10^6^ HCT116 or HT29 cells; tumor diameters were weekly tracked with an electronic caliper. Sera were collected for serological assays through retro-orbital puncture at day 0 and every week until the tumor diameter reaches 8 mm. At the end of a set of experiments, mice were sacrificed, blood was collected by intra-cardiac route, serum was isolated following centrifugation at room temperature and tumors were cryopreserved.

### Patients

Sera were collected from patients undergoing colonoscopy for digestive complaints or for screening. Six subjects had normal colonoscopy and were used as controls, fourteen patients had adenomatous polyps and nine had carcinoma; the mean ages of these three categories of patients were 71±4, 67±3 and 71±3 years, respectively. Blood was collected on neutral-type tubes and after centrifugation, serum was aliquoted and stored at −80°C.

### 2-Dimensional Electrophoresis Analysis and SERPA

For the extraction of proteins, normoxic or hypoxic cells were washed with 20 mM sodium phosphate-buffered saline (PBS) and scraped with DIGE labelling (DLA) lysis buffer (7 M urea, 2 M thiourea, 4% CHAPS and 30 mM Tris, pH 8.5). Supernatant was then recovered after centrifugation for 10 minutes at 10000 rpm and 4°C and concentration was determined by Bradford protein assay.

For SERPA experiments, 25 µg of protein extract was minimally labelled with 200 pmol of cyanine dye Cy5 (Amersham GE Healthcare) for 30 minutes in the dark on ice, according to the manufacturer’s protocol. Labelling reaction was stopped by incubating the mixture with 10 mM lysine (Sigma Aldrich) for 10 minutes. Non labelled proteins were added to reach a total of 250 µg of proteins and diluted in an appropriate loading buffer (4% 3-[(3-cholamidopropyl)dimethylammonio]-1-propanesulfonate (CHAPS), 7 M urea, 2 M thiourea, 30 mM Tris, 30 mM dithiothreitol (DTT), 1% IPG buffer 3–11 [GE Healthcare]). Samples were loaded onto rehydrated first dimension strip (Immobiline DryStrip, pH 3–11 NL, 18 cm, GE Healthcare). The samples were separated on 2 dimensional gel, using isoelectric focusing in the first dimension (300 V for 3 hours, gradient steps of 1000 V for 8 hours, 8000 V for 3 hours, and 8000 V for 45 minutes at 20°C with a maximum current settings of 50 µA per strip) and SDS polyarcrylamide gel (10% acrylamide) electrophoresis (SDS-PAGE) in the second dimension. The proteins were finally transferred onto a low-fluorescence PVDF membrane. Membranes were incubated for 2 h in 5% non-fat dry milk-containing Tris buffer saline with 1% of Tween (TTBS) blocking buffer and then exposed overnight at room temperature to either control or tumor-bearing mice serum in 1% non-fat dry milk-containing TTBS (dilution 1/100). For each experiment, a pool of sera collected from 6 different mice was used to ensure the robustness of the screening method. Immunodetection was performed using HRP-conjugated anti-mouse IgG secondary antibodies and ECL Plus reagent (GE Healthcare). Membranes were scanned at the Cy5 wavelength on a Typhoon FLA9500 Imager (GE Healthcare) for the detection of proteins and at the Cy2 wavelength for the detection of antibodies, exploiting the HRP-catalysed production of a fluorescent intermediate emitting at 503 nm from the Acridan substrate contained in the ECL Plus reagent. For validation of SERPA experiments, membranes were incubated with commercial antibody against eEF2 (Abcam, Cambridge, UK) and HRP-conjugated secondary antibodies (1/5000, Jackson Immunoresearch, Sufolk, UK).

### In Gel Enzymatic Digestion and Mass Spectrometry (MS) Identification

For sample recovery, unlabelled proteins were separated by 2D electrophoresis. The 2D gels were fluorescently-stained (Krypton protein stain, Pierce, Thermo Scientific) after fixation in a 50% water, 40% ethanol and 10% acetic acid solution, 1 h at room temperature. The proteins of interest were automatically picked from the gels using an Ettan Spot Picker (GE Healthcare). After rinsing, the 2D gel spots were dehydrated in acetonitrile at 37°C. Digestion was performed overnight at 37°C with trypsin (12.5 ng/ml) in 100 mM ammonium bicarbonate. The extraction step was performed with formic acid 5% for 15 min at 37°C**.** Supernatants were then used for the mass spectrometry identification of proteins with a nano-LC-ESI-MS/MS maxis 4G UHR-TOF (Bruker). Proteins of interest were identified thanks to Mascot software (Matrix Science, www.matrixscience.com). Then the NCBI nonredundant protein database was searched with mammals as taxonomy. Only significant hits, as defined by the Mascot probability analysis (p<0.001), were accepted and Protein scores >63 were considered statistically significant.

### Immunoblotting and Immunohistochemistry

Immunobloting and immunostaining were performed with antibodies against eEF2 (1/1000, Abcam), or phospho-Thr56-eEF2 (1/1000, Abcam). Gel loading was normalized with actin antibody (Sigma-Aldrich). Revelation was done with an anti-rabbit IgG antibody coupled with horseradish peroxidase (Jackson ImmunoResearch) and ECL Plus (GE). For immunochemistry, 5 µm sections of frozen xenografted HCT116 tumors were mounted on slides for immunostaining. Protein phosphatase (Protein Phosphatase, lambda, Calbiochem) was used according to the manufacturer’s protocol to validate the specificity of phosphorylated immunostaining. After treatment with phosphatase, tumor sections were probed with anti-eEF2 (1/50) or anti-Thr56 phosphorylated eEF2 (1/50) antibodies and were immunostained with anti-rabbit Alexa 488 (Invitrogen).

### eEF2 Immunoassay

Amounts of circulating antibodies directed against phosphorylated Thr56 eEF2 were determined in a dedicated immunoassay. 96-well plates (Reacti-Bind, Thermo Scientific) were coated overnight at room temperature with 10 µg/ml of a 12 amino acid phosphorylated peptide of eEF2. The phosphopeptide sequence was RAGETRFTDTRK, corresponding to amino acids 50 to 61 of the eEF2 protein, with a phosphorylation on Thr56 (Eurogentec). Coating and blocking steps were carried out using ELISA coating buffer and ELISA ultrablock (Abd Serotec) according to the manufacturer’s instructions. Diluted sera (1/100 for mice and 1/200 for humans) were incubated overnight at 4°C. After washing, specific hybridization was measured with a peroxidase-conjugated anti-mouse IgG antibody (dilution 1/10 000, Jackson ImmunoResearch) and addition of 3,3′,5,5′-tetramethylbenzidine (TMB, Calbiochem). Plates were read at 450 nm in a VictorX4 microplate reader.

### Statistics

Results are expressed as means ± s.e.m. Student’s *t* test and ANOVA tests were used where appropriate. *P<0.05, **P<0.01 or ***P<0.001 was considered statistically significant in the different experiments. For clinical data, sub-populations of patients were automatically detected by running the K-means clustering algorithm [29]; pairwise comparisons between distinct profiles within each condition were assessed according to a t-test including Benjamini-Hochberg FDR correction for multiplicity of the test [30].

## Results

### SERPA Identification of eEF2 aAb in the Serum of Tumor-bearing Mice

**Figure 1:**
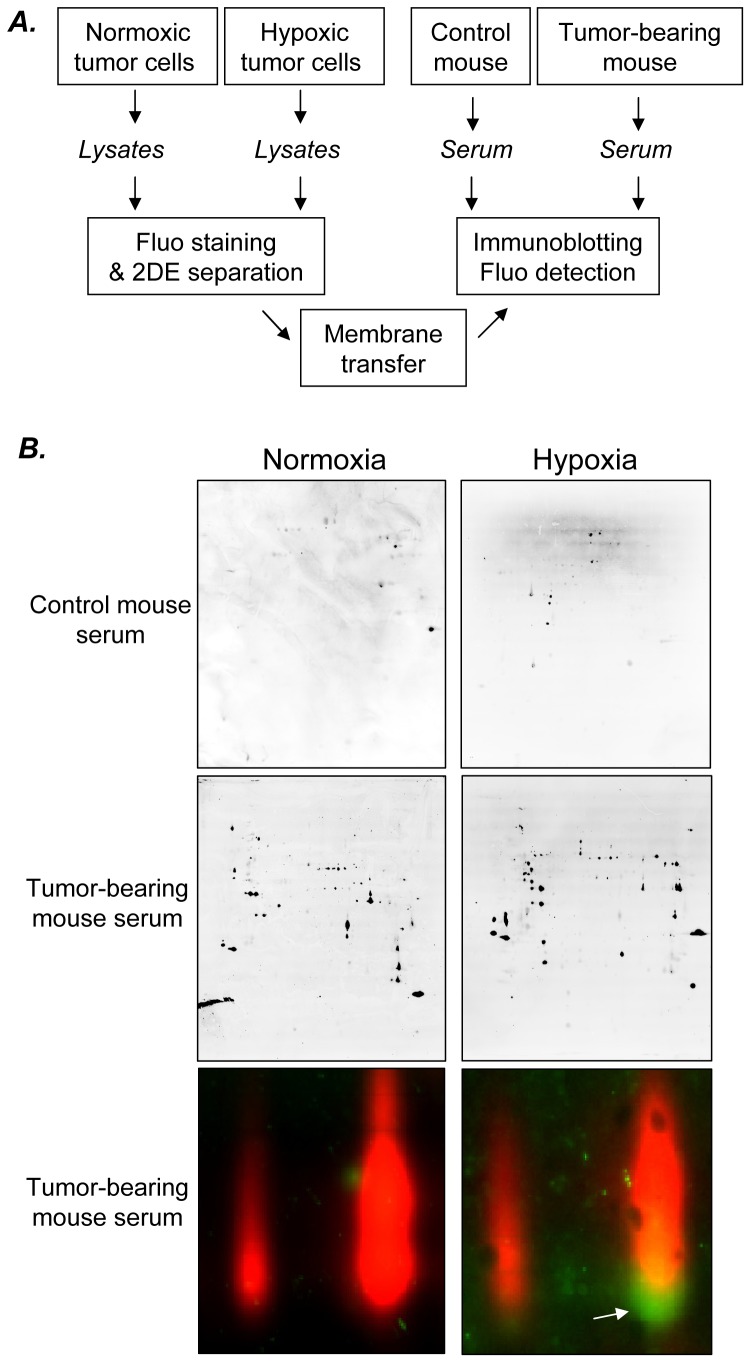
Hypoxia integration in the SERPA strategy. **A.** Workflow of the SERPA process including 2DE-gel separation of lysates from either hypoxic or normoxic tumor cells, membrane transfer, immunoblotting with the serum from either control or tumor-bearing mice, and detection of spots of interest. **B.** Typical immunoblotting patterns resulting from the incubation of 2D-resolved lysates of HCT116 cells exposed to normoxia or hypoxia, with the indicated mouse serum. In the bottom panels, proteins of the lysates are labelled with Cy dye (red) and fixed antibodies are detected with an anti-mouse secondary antibody (green spot); arrow indicates the presence of a protein exclusively detected in the lysates of hypoxic tumor cells by antibodies from the serum of tumor-bearing mice.

**Figure 2:**
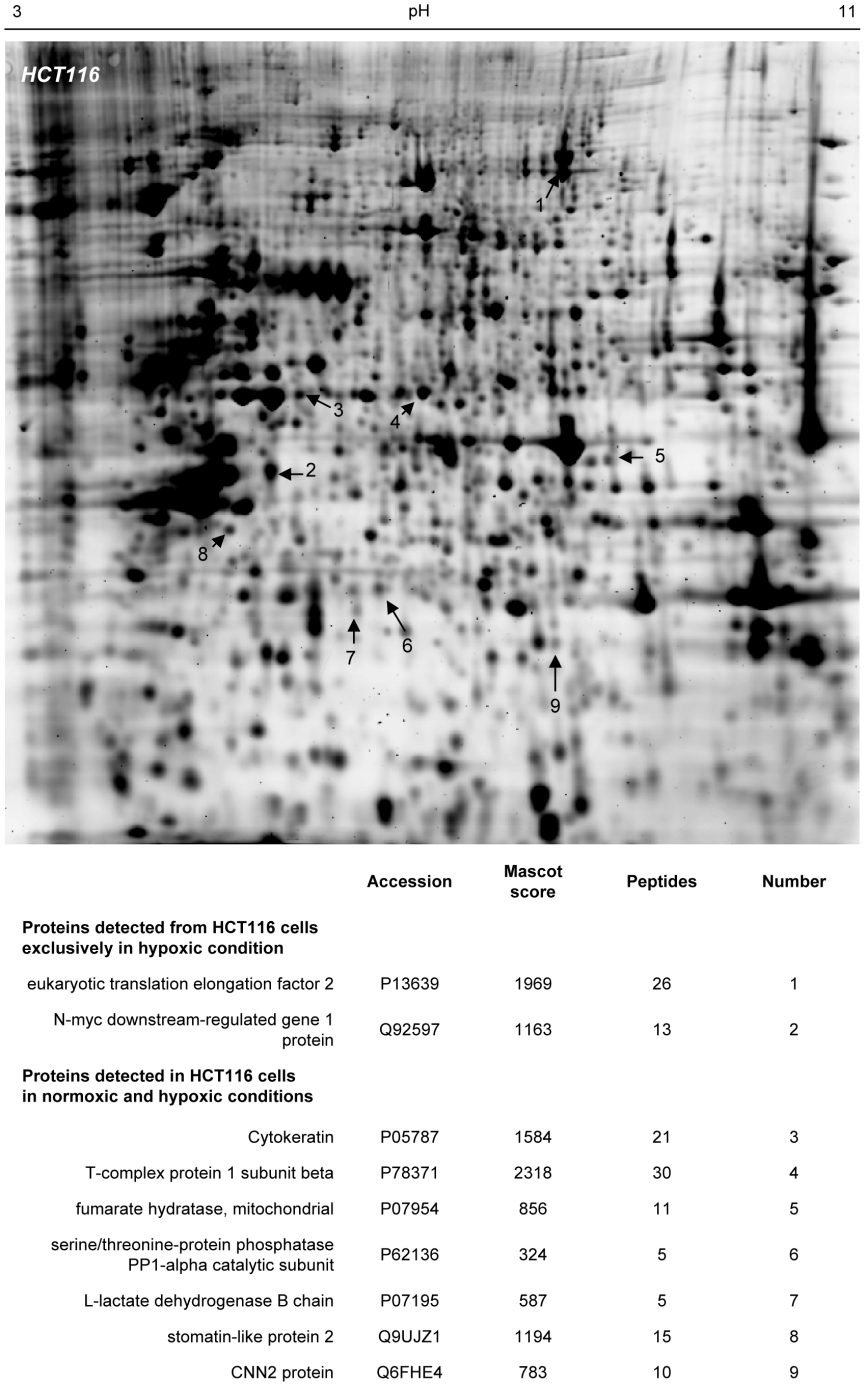
MS/MS-identification of proteins detected by SERPA from colorectal cancer cells exposed to hypoxia. Mapping of spots of interest resulting from the comparison described in Fig.1 and list of identified proteins (p<0.001) obtained using lysates of HCT116 colorectal cancer cells.

**Figure 3:**
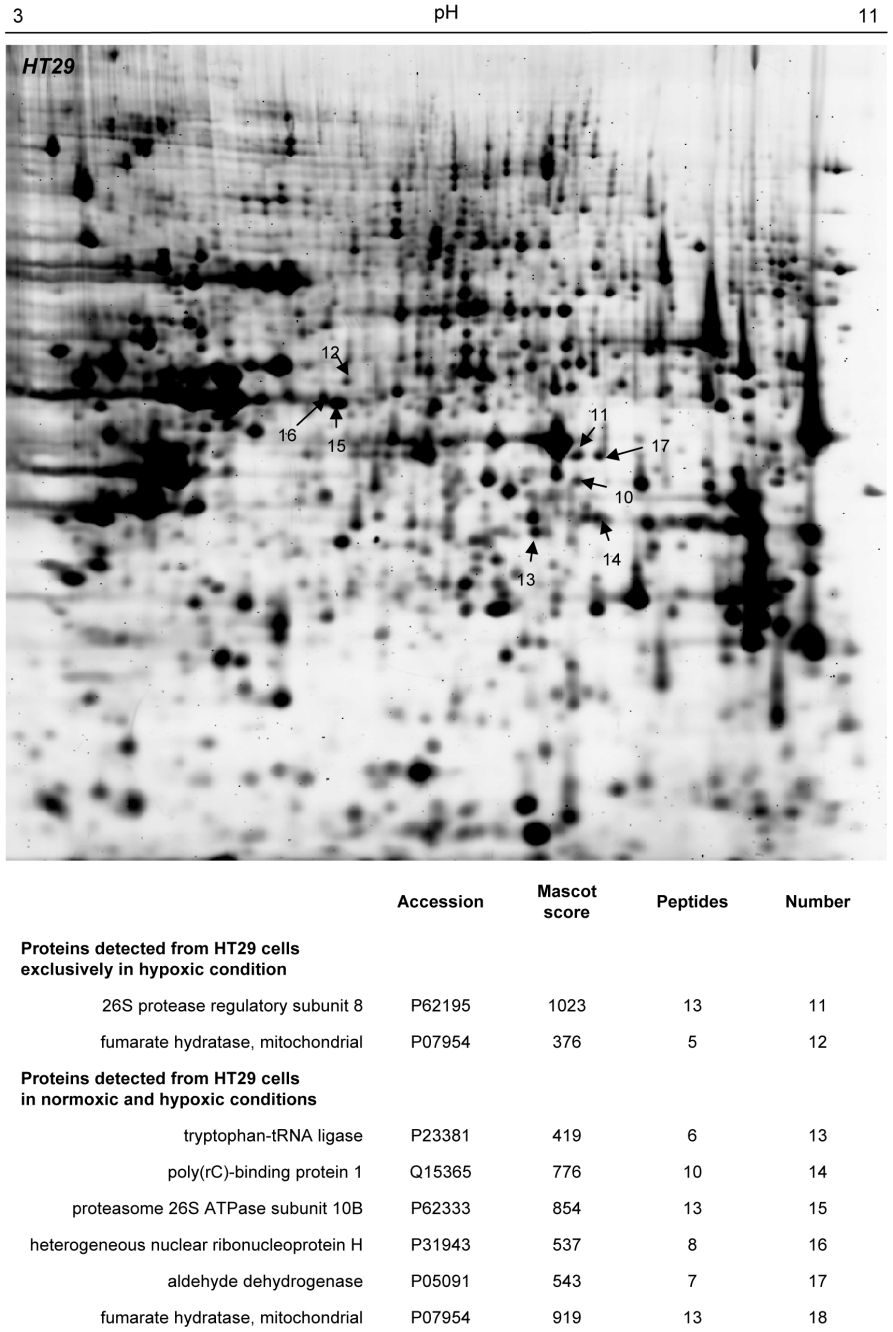
MS/MS-identification of proteins detected by SERPA from colorectal cancer cells exposed to hypoxia. Mapping of spots of interest resulting from the comparison described in Fig.1 and list of identified proteins (p<0.001) obtained using lysates of HT29 colorectal cancer cells.

To mimic the tumor microenvironment, the human colorectal HCT116 and HT29 cells were exposed to hypoxia (1% O_2_) for 48 hours, a time interval required for the expression of the hypoxia-inducible gene program at the protein level and for reaching a new equilibrium rate of tumor cell proliferation. Cells maintained under normoxic conditions (21% O_2_) were used as controls. We then applied the SERPA technology to identify hypoxia-specific tumor antigens (Figure 1A). HCT116 and HT29 lysates were first separated on gel by 2-dimensional electrophoresis and transferred onto membranes. Pooled sera from control or tumor-bearing mice (6 sera per condition) were then used to reveal spots corresponding to immunogenic proteins. Differential analysis was performed by comparing 4 different conditions: lysates from normoxic and hypoxic cells probed with sera from control and tumor-bearing mice (Figure 1B). This analysis allowed us to discard two types of spots: (i) those corresponding to proteins recognized by antibodies from control mouse sera and (ii) those corresponding to proteins recognized by antibodies from tumor-bearing mice but failing to be expressed under hypoxia. Remaining spots of interest were then excised from preparative gels, digested by trypsin and analyzed by MS (Figures 2 and 3). By combining both HCT116 and HT29 cell lines, we found 17 proteins with satisfying Mascot scores (p<0.001) which were recognized by antibodies from tumor-bearing mice, 4 hypoxia-specific proteins and 13 corresponding to proteins expressed both under hypoxic and normoxic conditions (see lower panels in Figures 2 and 3). For the rest of this study, we decided to focus on the hypoxia-specific antigen strictly reactive with the serum of tumor-bearing mice and identified by MS with the strongest Mascot score, namely eEF2 or eukaryotic elongation factor 2.

**Figure 4:**
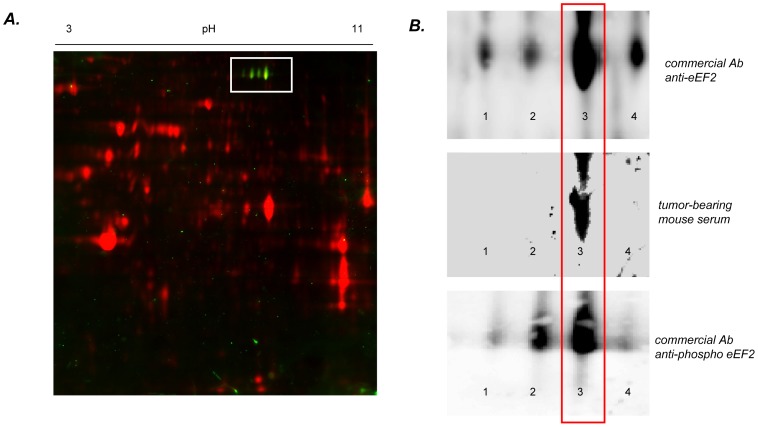
Validation of phospho-eEF2 protein as the target of autoantibodies in mice bearing colorectal HCT116 tumors. **A.** Representative immunoblotting of 2D-separated lysates of hypoxic HCT116 cells with a commercial antibody against eEF2. Proteins of the lysates are labelled with Cy dye (red) and secondary antibody is conjugated to horseradish peroxidase (green spots). Positive signal is obtained for several spots of the same molecular weight but differing by their pI value. **B.** Comparison of the eEF2 spots detected with a commercial antibody against total eEF2 (top), the serum from tumor-bearing mice (middle) and a commercial antibody against phospho-Thr56 eEF2 (bottom). Spot 4 (rightmost spot) corresponds to the unphosphorylated form of eEF2 while the other spots correspond to multi-phosphorylated forms of the protein; spot 3 (second spot from the right) corresponds to the preferential monophosphorylated form of eEF2 (on Thr56).

First, to validate the nature of the eEF2 protein present in the 2D gels, we probed the membrane with a commercially available anti-eEF2 antibody and found that the immunoblot signal matched the localization of the spot identified by the SERPA analysis (Figure 4A). Moreover, this experiment showed the distribution of the protein at different isoelectric points (Figure 4A and 4B, top panel). In the SERPA experiment however, one spot (the third one according to the pI value range) was immunogenic (Figure 4B, middle panel), strongly suggesting that a post-translational modification could confer the immunogenicity.

### Phosphorylation of Thr56 eEF2 after Hypoxia in Human Colorectal Cancer Cells

**Figure 5:**
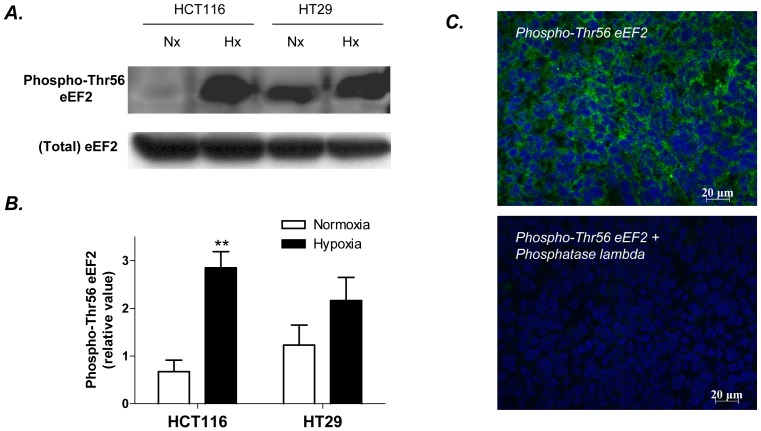
Validation of hypoxia-induced phosphorylations of eEF2 in colorectal cancer cells. **A.** Representative eEF2 and phospho-Thr56 eEF2 immunoblotting of HCT116 and HT29 cultured for 48 hours under hypoxia (Hx) or maintained in normoxia (Nx). **B.** Normalized expression of phospho-Thr56 eEF2 in normoxic vs hypoxic HCT116 and HT29 cells; n = 3, **p<0.01 **C.** Representative phospho-Thr56 eEF2 immunostaining of sections of HCT116 tumors in the absence (top) or the presence (bottom) of phosphatase lambda; note the complete disappearance of the phosphorylated form of eEF2 upon treatment with the phosphatase.

Since the phosphorylation of eEF2 is known to occur in response to hypoxia (leading to eEF2 inactivation and the arrest of protein translation), we examined the extent of eEF2 phosphorylation on Thr56 previously described as the first and main residue modified by a phosphate group within the eEF2 sequence [31]. Re-probing the 2D membrane used for SERPA confirmed that the spot recognized by the serum of tumor-bearing mice was also positively stained by a commercial anti-phospho-Thr56 eEF2 antibody (Figure 4B, lower panel). We also confirmed in conventional Western blotting experiments that eEF2 phosphorylation on Thr56 was significantly increased in hypoxic HCT116 cells (p<0.01) and that a similar trend was observed in HT29 cells (Figures 5A and 5B). Also, the injection of HCT116 cells into mice led to the development of a tumor with a robust staining of phospho-Thr56 eEF2 confirming the occurrence of this post-translational modification *in vivo* (Figure 5C, top panel). The treatment of tumor sections with phosphatase lambda completely abrogated the staining obtained with a commercial anti-phospho-eEF2 antibody (Figure 5C, bottom panel).

### Validation of Phospho-Thr56 eEF2 as a Tumor-associated Antigen and of the Corresponding aAb as Biomarker of Mouse Tumor Growth

**Figure 6:**
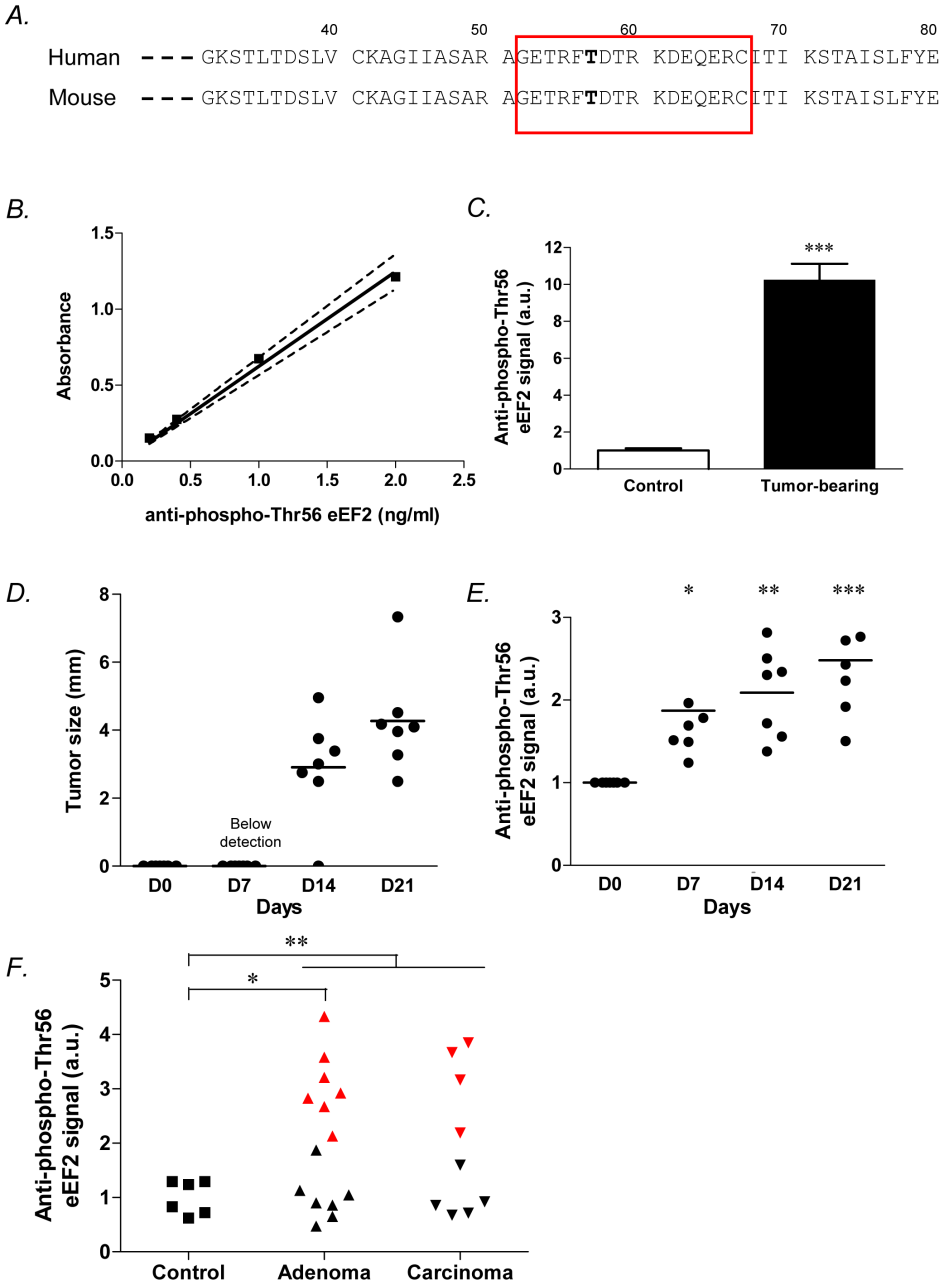
Changes in the titer of anti-phospho-eEF2 aAb as a marker of early tumor progression in mice and humans. **A.** Human and mouse amino acid sequences of eEF2 in the region of Thr56. The 12 residues corresponding to the synthetic peptide (phosphorylated on Thr56) used in our immunoassay are indicated (red frame); note the perfect identity between mouse and human sequences. **B.** Detection of commercial anti-phospho-Thr56 eEF2-antibodies using our immunoassay; dashed lines show the 95% confidence band of the linear regression. **C.** Detection of anti-phospho-Thr56 eEF2 aAb in the serum of control or HCT116 tumor-bearing mice (n = 3). ***P<0.001. **D.** Time course of HCT116 tumor growth as determined by measurements of tumor diameters (n = 7 per group). **E.** Detection of anti-phospho-Thr56 eEF2 aAb at the indicated time of HCT116 tumor progression. *P<0.05, **P<0.01, ***P<0.001 (n = 6–7 per group). Note that at day 7 post-implantation, tumors are not detectable (see panel D) but a positive signal is detected in the immunoassay. **F.** Graph represents the detection of anti-phospho-Thr56 eEF2 aAb in the serum of control subjects (n = 6) and patients with adenomatous polyps (n = 14) or carcinoma (n = 9). *P<0.05, **P<0.01. Of note, K-means clustering identified two subpopulations of patients (see black and red symbols) among individuals diagnosed with adenomatous polyps (P<0.001) and carcinoma (P<0.01); the same partition was observed in 100 independent runs by varying the random initialization of K-means algorithm.

To further explore the immunogenicity of the phospho-Thr56 eEF2, we developed an assay to probe the presence of aAb reactive against a phosphopeptide of 12 amino acids flanking Thr56, corresponding to amino acids 50 to 61 (Figures 6A). We validated the linearity of this immunoassay using the same commercial anti-phospho-Thr56 eEF2 antibody as described above (Figure 6B). In a first set of experiments, we used a pool of 6 sera of mice bearing large tumors (i.e., 28 days post-implantation) and found a 10-fold higher signal than when using control sera (Figure 6C). In a second set of experiments, we performed a time course study to determine the changes in phospho-Thr56 eEF2 signal according to the development of HCT116 tumors. Sera were collected by retro-orbital puncture in mice at day 0 and after 7, 14 and 21 days following the injection of HCT116 tumor cells; tumor growth was measured in parallel with an electronic caliper (Figure 6D). As shown in Figure 6E, the phospho-Thr56 eEF2 signal was already significantly increased at day 7 (p<0.05 *vs.* day 0) while at this time, the tumor was not yet detectable (Fig. 6D). At days 14 and 21, the extent of phospho-Thr56 eEF2 signal further increased in parallel to the growth of HCT116 tumors (Figures 6D and 6E).

### Anti-phospho Thr56 eEF2 Autoantibodies Identifiy Sub-populations of Patients with Colon Adenoma and Carcinoma

We finally examined the potential of the detection of anti-phospho-eEF2 antibodies to discriminate control subjects and patients with either adenomatous polyps or colorectal cacinoma. Because of the identity of sequences between mouse and human eEF2 in the residues flanking Thr56 (see Figure 6A), we used the same immunoassay for patients as the one described above for tumor-bearing mice. We found that patients diagnosed with adenomatous polyps and carcinoma showed an increase in the phospho-Thr56 eEF2 aAb titer (P = 0,0015) when compared with patients identified as negative following colonoscopy (Figure 6F). Moreover, when using the K-means clustering algorithm (29), two sub-populations of patients could be identified among the adenomatous polyps (P<0.001) and carcinoma groups (P<0.01) (see red symbols in Figure 6F).

## Discussion

The two major findings of this study are (i) that hypoxia accounts for the modification of the immunoproteome as evidenced by the detection of circulating aAb directed against TAA undetectable in tumor cells cultured under normoxic conditions, and (ii) that the hypoxia-mediated stimulation of eEF2 phosphorylation accounts for the development of an early aAb response to colorectal cancer development.

aAb are nowadays recognized as potential cancer biomarkers and SERPA was developed to detect them from serum specimens through the blotting of 2DE-separated tumor cell lysates. The SERPA technology, in contrast to SEREX and phage display, enables the detection of proteins that have undergone post-translational modifications. However, the proteome in lysates isolated from tumor cells cultured in a conventional incubator under normoxia is far from representing the proteome of tumor cells in their *in vivo* microenvironment. In particular, hypoxia is a hallmark of many cancers resulting from disequilibrium between O_2_ consumption and O_2_ availability in poorly vascularized tumors. The impact of hypoxia on the tumor cell transcriptome and proteome is double: while the global translational machinery is slowed down to spare energy, specific gene programs regulated by key transcription factors such as the HIF family, are induced to allow tumor cell adaptation [6]. Importantly, in epithelial cancers such as the colorectal cancer, hypoxia is also proposed to occur early during carcinogenesis. Mutant cells are indeed initially separated from the underlying blood vessels by the still intact basement membrane: this leads to the development of premalignant lesions in avascular regions and heading towards the opposite, less-constrained regions [11].

In the current study, we therefore used two analytical filters to select potential TAA for further validation. First, we excluded spots identified on 2D membranes after immunoblotting with serum collected from control mice. Second, we did not consider for picking the proteins detected by the serum of tumor-bearing mice but only expressed in normoxic tumor cells. This strategy allowed to reduce the number of false-positive results and to favor the detection of specific TAA as encountered in *in vivo* conditions. We used two different colorectal cancer cell lines (P53-wild-type HCT116 and P53-mutant HT29) to further increase the diversity of the proteome, and in particular of the immunoproteome. This strategy led to the identification of a total of 17 putative TAA, 13 expressed under both hypoxia and normoxia and 4 being exclusively expressed under hypoxia (see figures 2 and 3). Among the latter, we focused on the eukaryotic translation elongation factor 2 (eEF2), an essential factor for ribosomal mRNA translation. Hypoxia is known to promote eEF2 inactivation to block the high energy-consuming protein synthesis process in order to spare energy [9]. Inactivation of eEF2 results from its phosphorylation on Thr56 by the eukaryotic elongation factor 2 kinase (eEF2K) through a variety of mechanisms involving mTOR, AMPK and PHD2 [32]–[34]. We actually identified this phosphorylated form of eEF2 as the immunogenic protein. Although several phosphorylation sites are reported for eEF2 as evidenced by the four spots with distinct pI detected by a total eEF2 antibody, the location of the positive spot in SERPA pointed phospho-Thr56 eEF2 as the seroreactive entity. The second position from the right is indeed compatible with the preferred phosphorylation site previously reported to be Thr56 in a kinetic study on the regulation of eEF2 [31]. We then confirmed by using an immunoassay with a modified peptide phosphorylated on the Thr56 residue, that this region accounted for the immunogenicity of eEF2 as detected in our SERPA study. Using this assay, we found that anti-phospho-Thr56 eEF2 aAb were detectable in the mouse serum before tumors could be palpable. Also, we found that these aAb could be detected in humans with adenomatous polyps and colorectal cancers. Interestingly, in these two groups, patients could be statistically clustered in two subpopulations. In patients with adenomatous polyps, the K-means clustering of individuals with a high seric titer of anti-Thr56 eEF2 antibody could be related to a higher potential of cancer progression. Of note, in carcinoma patients, a distinct reactivity of aAb according to the time-to-diagnosis was recently proposed to relate to the formation of immune complexes with circulating TAA, thereby leading to plasma depletion of free aAb [35]. Our results therefore warrant further evaluation of anti-phospho-Thr56 eEF2 aAb as a potential diagnostic and possibly prognostic biomarker of colorectal cancer using large cohorts of patients at different stages of the disease. This objective is particularly attractive considering the data obtained in patients with adenomatous polyps since today, the determination of their potential of evolution towards carcinoma requires repetitive colonoscopies with endoscopic resection for histological analysis. Blood screening for the presence of biomarkers such as anti-phospho-Thr56 eEF2 aAb could contribute to the stratification of patients in risk groups for tailored colorectal cancer prevention programs.

This study also provides the demonstration that although performed in nude mice exhibiting a limited humoral immune response, this experimental setup remains an accessible methodology to obtain candidates biomarkers for further validation with human serum samples. Importantly, however, the specificity of phospho-Thr56 eEF2 aAb as cancer biomarker will have to be addressed. Indeed, hypoxia is involved in other pathological situations than cancer, including diabetes, atherosclerosis and chronic obstructive pulmonary disease. Also, circulating aAbs directed against eEF2 kinase were reported in patients with systemic lupus erythematosus [36] and such response involving eEF2 kinase and possibly eEF2 itself could therefore represent a more general response against stress-related and/or starvation-related conditions.

In conclusion, this study provides evidence that mimicking the *in vivo* microenvironment may unmask the presence of autoantibodies directed against proteins normally not present in the proteome of tumor cells cultured under normoxia. Furthermore, the identification of phospho-eEF2 as an immunogenic entity giving rise to the production of autoantibodies in patients with adenomatous polyps and colorectal cancers indicates that valid post-translational modifications may also be recapitulated with this strategy. This strategy opens new perspectives for the use of SERPA and other proteomics-based strategies to identify aAb as *bona fide* markers of carcinogenesis and early tumor progression.
